# Using Large Language Models to Support Content Analysis: A Case Study of ChatGPT for Adverse Event Detection

**DOI:** 10.2196/52499

**Published:** 2024-05-02

**Authors:** Eric C Leas, John W Ayers, Nimit Desai, Mark Dredze, Michael Hogarth, Davey M Smith

**Affiliations:** 1 Herbert Wertheim School of Public Health and Human Longevity Science University of California San Diego La Jolla, CA United States; 2 Qualcomm Institute University of California San Diego La Jolla, CA United States; 3 Division of Infectious Diseases and Global Public Health Department of Medicine University of California San Diego La Jolla, CA United States; 4 Altman Clinical Translational Research Institute University of California San Diego La Jolla, CA United States; 5 Department of Computer Science Johns Hopkins University Baltimore, MD United States; 6 Department of Biomedical Informatics University of California San Diego La Jolla, CA United States

**Keywords:** adverse events, artificial intelligence, AI, text analysis, annotation, ChatGPT, LLM, large language model, cannabis, delta-8-THC, delta-8-tetrahydrocannabiol

## Abstract

This study explores the potential of using large language models to assist content analysis by conducting a case study to identify adverse events (AEs) in social media posts. The case study compares ChatGPT’s performance with human annotators’ in detecting AEs associated with delta-8-tetrahydrocannabinol, a cannabis-derived product. Using the identical instructions given to human annotators, ChatGPT closely approximated human results, with a high degree of agreement noted: 94.4% (9436/10,000) for any AE detection (Fleiss κ=0.95) and 99.3% (9931/10,000) for serious AEs (κ=0.96). These findings suggest that ChatGPT has the potential to replicate human annotation accurately and efficiently. The study recognizes possible limitations, including concerns about the generalizability due to ChatGPT’s training data, and prompts further research with different models, data sources, and content analysis tasks. The study highlights the promise of large language models for enhancing the efficiency of biomedical research.

## Introduction

Biomedical text analysis is commonly burdened by the need for manual data review and annotation, which is costly and time-consuming. Artificial intelligence (AI) tools, including large language models (LLMs) such as ChatGPT (OpenAI) [1], could reduce this burden by allowing scientists to leverage vast amounts of text data (including medical records and public data) with short written prompts as annotation instructions [2]. To explore the potential for AI-assisted annotation, we evaluated whether ChatGPT could replicate human identification of adverse events (AEs) about a cannabis-derived product (delta-8-tetrahydrocannabinol) reported in social media posts [3]. AE detection requires reviewing a large amount of unstructured text data to flag a tiny fraction of AE reports, making it an ideal application for AI-assisted annotation [4].

## Methods

### Overview

To reduce selective reporting bias, we replicated a peer-reviewed publication, wherein human annotators identified AEs in 10,000 randomly sampled, publicly available posts from a delta-8-tetrahydrocannabiol social media forum (Reddit’s r/delta8) [3]. Human annotators identified potential AE reports (yes or no) and whether the AE was serious according to 6 Food and Drug Administration MedWatch categories (eg, hospitalization) [5].

ChatGPT (gpt-3.5-turbo-0613) was set to the default settings (*Temperature*=1, *Top P*=1, *Max token limit*=1700, *Frequency Penalty*=0, and *Presence Penalty*=0); given each Reddit post; and asked to reference annotation instructions identical to those given to human annotators, except for a minor modification for result formatting (ie, requested codes in a comma-delimited format; Multimedia Appendix 1). Since ChatGPT was treated as an additional annotator, we compared ChatGPT’s responses with human annotations using the traditional method for assessing interrater reliability rather than statistics for assessing classifiers (eg, *F*_1_-score). Thus, we calculated absolute agreement and prevalence- and bias-adjusted Fleiss κ statistics for any AEs, serious AEs, and each MedWatch category of serious AEs [6]. Analyses were computed with R statistical software (version 4.3.1; R Core Team).

### Ethical Considerations

This study was exempted by the University of California San Diego’s human research protection program because the data were public and nonidentifiable (45 CFR §46).

## Results

ChatGPT returned misformatted responses (eg, including the text “adverse event” instead of the requested “0” or “1”) in 35 (0.35%) of 10,000 instances. All misformatted responses were interpretable and resolved through normal data-cleaning methods (eg, rule matching). Example posts along with their labels are shown in Table 1. ChatGPT and human annotators agreed on 94.4% (9436/10,000) of labels for any AEs (κ=0.95) and 99.3% (9931/10,000) of labels for any serious AEs (κ=0.96; Table 2). For serious AEs, the lowest agreement was 99.4% (9939/10,000) for “other” serious (but undefined) outcomes (κ=0.98). All specifically defined outcomes (eg, hospitalization) achieved 99.9% (≥9986/10,000) agreement (κ=0.99).

**Table 1:** Example of posts to the Reddit community r/delta8 and the corresponding categorizations.

Title and text	Labels^a^
Had to be rushed to the ER after eating an edible. Last week me and my boyfriend bought delta 8 edibles from a vape shop. We were bored and decided it would be a good idea to test it out, we ate two (approximately .1 gram in total). Just a side note, this is was not my first time eating an edible so I didn't really think much of it. It took about 40 minutes for the edible to kick in, at first I just felt very heavy and It was super hard to move, so I laid down for about an hour. Eventually I got bored of laying down and got up to go shower...bad decision. According to my boyfriend, when I got up I fainted. I remember waking up to him freaking tf out, it was very hard to breathe, and it felt like my heart was going to burst. They rushed me to the ER because I was barely able to stay conscious. I had a phycotic break, I thought I was dead, kept hearing all kinds of noises, and I completely lost touch with reality. My heart rate was over 165, I also have a heart condition so they had to keep an eye on that too. It was the most terrifying and traumatizing experience, and I'm still not over it yet. Has anyone gone through this before?	Identified as an adverse event report and considered serious with the following outcomes: life-threatening, hospitalization, and other serious adverse event
Help I feel hungover from delta 8. I feel so awful and can't stop puking. I took 10 mg last night and still feel horrible today. Any advice?	Identified as an adverse event report, but not considered serious
Battery Question. Can someone please recommend and ideal wattage/voltage to use the [BRAND] with? I only have variable wattage/voltage batteries for nicotine vaping and am unfamiliar with batteries used for oils. I’m assuming the former type should work fine as long as I have them set low enough? Any help is appreciated. Thanks	Not identified as an adverse event report

^a^Serious adverse events were defined using the Food and Drug Administration MedWatch health outcome categories, which include life-threatening; hospitalization; disability or permanent damage; congenital anomaly or birth defect; required intervention to prevent permanent impairment; or other serious event.

**Table 2:** Accuracy of ChatGPT in replicating human identification of adverse events in r/delta8 posts (N=10,000) and the categorization of adverse events to the Food and Drug Administration MedWatch outcome categories.

MedWatch categories and ChatGPT response		Human annotation			Agreement, n (%)			κ statistic^a^	
		Yes, n	No, n						
**Labeled as an adverse event report**						9436 (94.4)			0.95
	Yes	172	401						
	No	163	9264						
**Labeled as a serious adverse event report^b^**						9331 (99.3)			0.96
	Yes	15	17						
	No	52	9916						
**Life-threatening**						9995 (99.9)			0.99
	Yes	1	5						
	No	0	9994						
**Hospitalization**									
	Yes	5	6	9993 (99.9)			0.99		
	No	1	9988						
**Disability or permanent damage**						9998 (99.9)			N/A^c^
	Yes	0	2						
	No	0	9998						
**Congenital anomaly or birth defect**						9999 (99.9)			N/A
	Yes	0	1						
	No	0	9999						
**Required intervention to prevent permanent impairment or damage**						9986 (99.9)			0.99
	Yes	0	2						
	No	12	9986						
**Other serious or important medical events**						9939 (99.4)			0.98
	Yes	7	13						
	No	48	9932						

^a^Prevalence- and bias-adjusted Fleiss κ.

^b^A composite of any of the 6 adverse event outcomes.

^c^N/A: not applicable (κ could not be calculated due to no events being found by human annotators).

## Discussion

ChatGPT demonstrated near-perfect replication of human-identified AEs in social media posts using the exact instructions that guided human annotators. Despite significant resource allocation, automating AE detection has seen limited success. Many studies (eg, social media studies) often omit performance metrics such as agreement with ground truth altogether [7]. The LLM and prompt used outperformed the best-performing specialized software for detecting AEs from text data (agreement=94.5%; κ=0.89), which relied on structured and human-curated electronic discharge summaries [8].

We note a few limitations. First, we did not have any measures from the replicated study to estimate time or cost savings attributable to using an LLM. However, these savings would be considerable. If a human annotated 1 post/min, the replicated study’s estimated completion time would be 166.6 hours (10,000 posts × 60 posts/h), or 20.8 workdays. Conversely, assuming ChatGPT annotated a post in 2 seconds [9], it would take 5.6 hours with no human effort. Second, the social media data analyzed may be included in ChatGPT’s underlying training data, potentially inflating the accuracy reported herein and reducing generalizability. Third, our goal was to replicate human annotation using the exact codebook that trained human annotators and default settings of ChatGPT-3.5-turbo. Although this alone showed promise, further improvements to the prompt, different models (GPT-4 or Llama-2), or alternative model parameter specifications may improve the accuracy. Finally, we only assessed 1 application of an LLM for biomedical text analysis; inaccuracy and label bias may exist in other settings. Further research is needed to capture process outcomes (eg, time savings), apply LLMs to traditional biomedical data (eg, health records), and address more complex methods of annotation (eg, open coding).

While acknowledging its limitations, this case study demonstrates the potential for AI to assist researchers in text analysis. Given the demand for annotations in biomedical research and the inherent time and cost constraints, adopting LLM-powered tools could expedite the research process and consequently scientific discovery.

## Appendix

Multimedia Appendix 1 Prompt used to train ChatGPT.

## Abbreviations

AE: adverse event
AI: artificial intelligence
LLM: large language model

## Data Availability

The corresponding data for the study are available on the first author’s website [10].
