# Supplementary material for: Using Large Language Models to Support Content Analysis: A Case Study of ChatGPT for Adverse Event Detection
Source: J Med Internet Res. 2024 May 2;26:e52499. doi: 10.2196/52499 (PMC11099800; doi:10.2196/52499)
Supplement: Multimedia Appendix 1 [file jmir_v26i1e52499_app1.docx]

**Multimedia Appendix 1. Prompt used to train ChatGPT.**

†Read the post carefully and identify whether the post contains any of the following variables. If the post contains the variable please return a 1. If the post does not contain the variables, return a 0. Please return output as a comma delimited string with 0s or 1s for coded responses to all 7 variables.*

Please check both the self-text and title for information. If the self-text is unavailable or says 'removed' or 'N/A,' check the title to see if it indicates an adverse event from the author's own delta-8-THC use.

For all variables please only record an event describing an author's personal use of delta-8-THC. Please consider the following rules related to personal delta-8-THC use: a. Interpret all nicknames and variations of delta-8-THC (such as 'Delta-8,' 'd8,' 'Date,' 'Δ8,' 'delta-8-THC', 'delta') as references to delta-8-THC. b. Exclude discussions about other substances unless the author explicitly describes their use of delta-8-THC in addition to the other substances. c. Only consider use when the author explicitly mentions using delta-8-THC and not when discussing purchasing, possession, or finding delta-8-THC but not use. d. Be cautious with verb tenses; delta-8-THC use must be current or have occurred in the past, but asking questions about delta-8-THC do not count unless the author also states they have used delta-8-THC. e. Common brand names alone do not indicate delta-8-THC use, unless the author mentions using a delta-8-THC product from that brand. g. When in doubt, default to being conservative and select 0 for delta-8-THC use.

Any adverse event

Check this option if: the author is reporting experiencing any undesirable clinical experience associated with personal use of delta-8-THC, including unfavorable signs or symptoms, unusual lab results, new illness or disease, or deterioration of existing conditions.

Do not check this option if: a. The event occurred to another individual described but not the author. b. This is not about the author's personal health, including mental health, physical health, perceived symptoms, and diagnosed symptoms. c. The event is only about negative experiences with delta-8-THC (e.g., malfunctioning) but not a health problem. d. The adverse event is not related to an author's delta-8-THC use. e. When in doubt, default to selecting 0 for adverse health events related to delta-8-THC.

Life-threatening

Check this option if: the author was at substantial risk of dying at the time of the adverse event, or use or continued use of delta-8-THC might have resulted in the death of the author. If the author is describing that they 'feel' as if they are dying or 'thought' they were going to die, that does not count here. If they are at risk of dying return a 1, if not return a 0.

Do not check this option if: The author is describing that they 'feel' as if they are dying or 'thought' they were going to die. That does not count here.

Hospitalization (initial or prolonged)

Check this option if:

Admission to the hospital or prolongation of hospitalization was a result of the adverse event occurring from the authors use of delta-8-THC.

An author is admitted to the hospital for one or more days because the author used delta-8-THC, even if released on the same day.

An emergency room visit stemming from the use of delta-8-THC resulted in admission to the hospital.

Do not check this option if:

The author reported visiting the emergency room because of an adverse event related to delta-8-THC use, but the visit did not result in being admitted to the hospital.

An author in the hospital received a medical product and subsequently developed an otherwise non-serious adverse event, unless the adverse event prolonged the hospital stay.

If the author is only asking if they should seek medical care.

Disability or Permanent Damage

Check this option if: An adverse event related to delta-8-THC use resulted in a substantial disruption of a person's ability to conduct normal life functions. Such would be the case if the adverse event resulted in a significant, persistent or permanent change, impairment, damage or disruption in the author's body function/structure, physical activities and/or quality of life.

Do not check this option if: The author describes they are unable to move from being too high for a short period of time because of delta-8-THC use or any other acute symptom.

Congenital Anomaly/Birth Defects

Check this option if: The author suspects that delta-8-THC use prior to conception or during pregnancy may have resulted in an adverse outcome in the child.

Required Intervention to Prevent Permanent Impairment or Damage

Check this option if: The author believes that medical or surgical intervention was necessary to preclude permanent impairment of a body function or prevent permanent damage to a body structure, either situation suspected to be due to the use of delta-8-THC. For this, we can make the assumption that if the author sought medical attention at urgent care, emergency room, etc. for the adverse event, it implies that the event required medical intervention. However, to be classified as 'hospitalization,' the author must have been admitted to the hospital for at least one day.

Other Serious or Important Medical Events

Check this option if:

The event does not fit the other variables, but the event could have jeopardized the author and could have required medical or surgical intervention (treatment) to prevent one of the other outcomes and is due to delta-8-THC use.

Examples include a serious problem with breathing, serious blood disorders, or seizures/convulsions that do not result in hospitalization.

The development of drug dependence or drug abuse, including withdrawal symptoms, would also be examples of important medical events related to delta-8-THC use.

†Again, please return output as a comma delimited string with 0s or 1s for coded responses to all 7 variables.

† Denotes a paragraph that was edited to match ChatGPT output formatting.
